# Extraction, Isolation, Characterization, and Bioactivity of Polypropionates and Related Polyketide Metabolites from the Caribbean Region

**DOI:** 10.3390/antibiotics12071087

**Published:** 2023-06-22

**Authors:** Raúl R. Rodríguez-Berríos, Agnes M. Ríos-Delgado, Amanda P. Perdomo-Lizardo, Andrés E. Cardona-Rivera, Ángel G. Vidal-Rosado, Guillermo A. Narváez-Lozano, Iván A. Nieves-Quiñones, Jeremy A. Rodríguez-Vargas, Keiry Y. Álamo-Diverse, Naiara Lebrón-Acosta, Nataniel Medina-Berríos, Patricia S. Rivera-Lugo, Yeriel A. Avellanet-Crespo, Yermarie W. Ortiz-Colón

**Affiliations:** Department of Chemistry, University of Puerto Rico, Río Piedras Campus, P.O. Box 23346, San Juan PR 00931-3346, Puerto Rico

**Keywords:** antimicrobial, Caribbean Sea, Gulf of Mexico, linear polyketides, secondary metabolites, macrolides, natural products, polyethers

## Abstract

The Caribbean region is a hotspot of biodiversity (i.e., algae, sponges, corals, mollusks, microorganisms, cyanobacteria, and dinoflagellates) that produces secondary metabolites such as polyketides and polypropionates. Polyketides are a diverse class of natural products synthesized by organisms through a biosynthetic pathway catalyzed by polyketide synthase (PKS). This group of compounds is subdivided into fatty acids, aromatics, and polypropionates such as macrolides, and linear and cyclic polyethers. Researchers have studied the Caribbean region to find natural products and focused on isolation, purification, structural characterization, synthesis, and conducting biological assays against parasites, cancer, fungi, and bacteria. These studies have been summarized in this review, including research from 1981 to 2020. This review includes about 90 compounds isolated in the Caribbean that meet the structural properties of polyketides. Out of 90 compounds presented, 73 have the absolute stereochemical configuration, and 82 have shown biological activity. We expect to motivate the researchers to continue exploring the Caribbean region’s marine environments to discover and investigate new polyketide and polypropionate natural products.

## 1. Introduction

**Figure 1:**
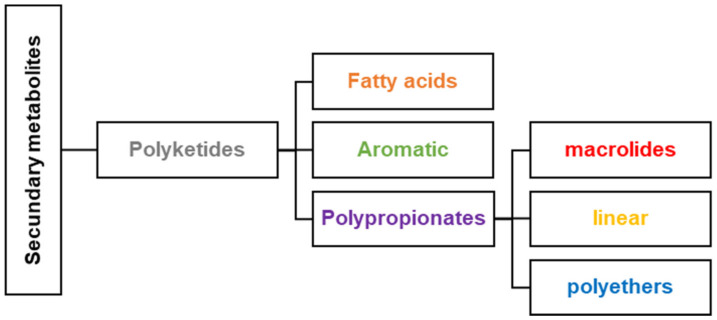
Classification of polyketides in natural products.

**Figure 2:**
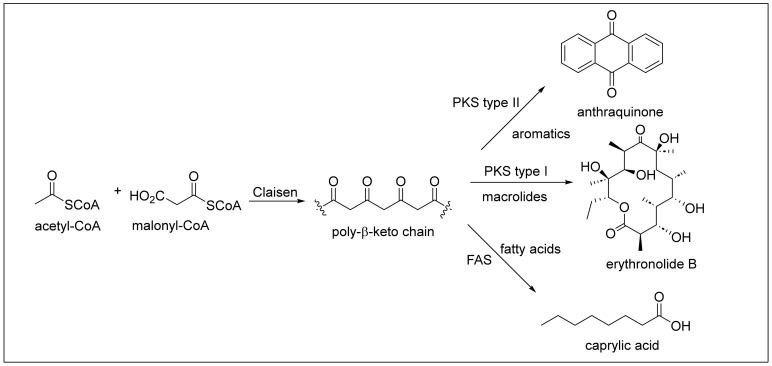
General diagram for the biosynthesis of polyketides.

The Caribbean region, often called the Caribbean or West Indies, is an area located around the Caribbean Sea and the Gulf of Mexico. The Caribbean coastal area stretches along the southern coast of the United States, the eastern coast of Central America, and the northern coast of South America. The Caribbean region is renowned for its rich marine biodiversity that produces secondary metabolites. Secondary metabolism involves a series of metabolic pathways the living organism uses to produce secondary metabolites or natural products. They have limited distribution and are unique in each species or family of organisms [1]. The biosynthesis of a natural product is different from one organism to another since it starts from other precursors or intermediates derived from the primary metabolism. Secondary metabolites, in organisms, play a crucial defense role against predators, parasites, and diseases, for competition and to facilitate the reproductive processes (coloring, odors, etc.). Polyketides are a broad class of secondary metabolites divided into fatty acids, polypropionates, and aromatic compounds (Figure 1). The biosynthesis of polyketides by animals, bacteria, fungi, and plants is derived from the acetate pathway through the Claisen condensation reactions of acetyl-CoA with malonyl Co-A (C_2_-units) to obtain poly-β-keto chains (Figure 2). This reaction produces poly-β-keto chains (Figure 2) catalyzed by the multifunctional enzymes polyketide synthases (PKSs) [2]. In this review, we will focus on the discussion of the polypropionates group that can be classified into subgroups, including macrolides, and linear and cyclic polyethers (Figure 2) [3,4]. Type I PKS is responsible for polypropionate macrolide biosynthesis, where the Claisen condensation employs either acetyl-CoA or, normally, propionyl-CoA (C_3_-units), or both [1]. The carbon chains for polypropionates are characterized by having partial, complete, or no reduction of the keto backbone. The aromatic compounds are biosynthesized by type II PKS, and no reduction of the keto backbone occurs. Depending on the organism, it is possible to combine more than one type of PKS and fatty acid synthase (FAS) protein to produce a variety of polyketide systems. Caribbean polyketide/polypropionate metabolites such as macrolides, linear polypropionates, and cyclic polyethers described in this review have been isolated principally from marine sponges and cyanobacteria, suggesting that these Caribbean organisms employ type I PKS enzymes for their biosynthesis (Figure 2). Only about 1% of the reported polyketides possess drug activity (typically antibiotic, anticancer, antifungal, antiparasitic, immunomodulatory action, and cholesterol-lowering agents). These account for approximately five times more bioactivity than average for other natural product families [5]. Around 20% of top-selling small-molecule drugs are polyketides [5]. As an example, marine invertebrates have been a prosperous source of secondary antimitotic polypropionates that show promising anticancer activity, including discodermolide (**12**), dolastatin-10, laulimalide, eleutherobin, halichondrin B, pelorusside, vitilevuamide, spongistatin, and hemiasterlin [6,7].

A review of polypropionate was reported by M. T. Davies-Coleman and M. J. Garson in 1998, which summarized that 168 marine polypropionates had been isolated from mollusks around the world [8]. Recently, in 2020, Zhang et al. summarized by structural features and biological activities 165 marine polypropionates reported from 1999 to 2020 [9]. A. Demeritte and W. M. Wuest, in 2020, reported a general review of a small selection of different kinds of Caribbean secondary metabolites, including polyketides, peptides, alkaloids, terpenes, and glucolipids [10]. One of the most recent and extended reviews summarizes all of the marine natural products reported in the literature during 2019 [11]. Around 1490 of all types of natural products were described by source organisms.

This review will present the isolation, classification, structures, and bioactivities of around 90 polyketide and polypropionate natural products collected around the Caribbean region in past years. For each polypropionate/polyketide metabolite, we described the organism source, what part of the Caribbean it was collected from, the purification methodology, and physical and chemical characterization methods. The purpose of this work is to spotlight the Caribbean region as a biodiverse place to find different organisms (marine or terrestrial) that produce bioactive polyketide products that will be used in the future as anticancer drugs, antimicrobial agents, and treatments for different kinds of diseases.

### 1.1. Extraction, Isolation, Structural Characterization, and Bioactivity

#### 1.1.1. Macrolides

**Figure 3:**
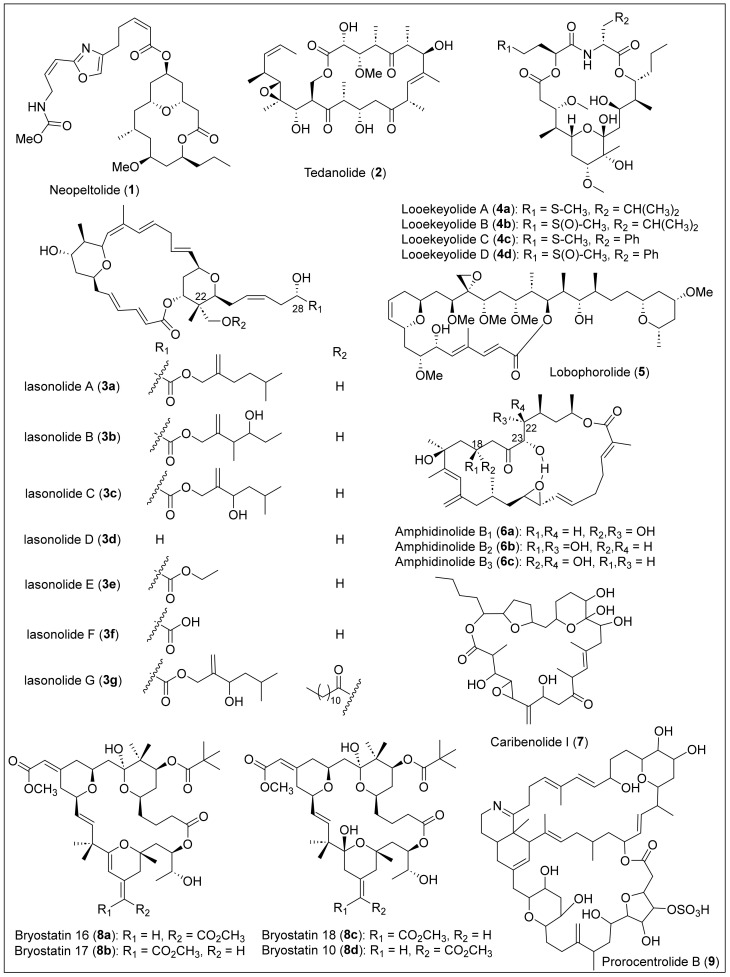
Structures of Caribbean macrolides **1**–**9**.

Macrolides are a large family of macrocyclic lactones and lactams composed primarily of propionate units or a mixture of acetate and propionate. Most exhibit antibiotic activity, typically with a ring size of 12 to 20 atoms. For example, erythronolide B (Figure 2) is a 14-membered macrolide antibiotic produced by *Saccharolyspora erythraea* and was isolated in 1952 [1]. Erythromycin is a commercially available antibiotic used against gram-positive bacteria and penicillin-resistant *Staphylococcus* strains to treat infections of *Legionella pneumophilia* [1]. Neopeltolide (**1**) is a bioactive 14-membered macrolide native from the Caribbean deep-sea sponge *Neopeltidae* from the genus *Daedalopelta*, collected from Jamaica’s north coast (Figure 3) [12]. For the isolation and purification of macrolide **1**, the sponge samples were obtained and stored at −20 °C. This sample was then extracted with ethanol. Later, it was subjected to concentration by distillation under reduced pressure and was partitioned between butanol and water. The butanol partition was further separated by vacuum-column chromatography using silica gel and ethyl acetate (EtOAc) in heptane to obtain a fraction of the sample. Neopeltolide (**1**) was purified using reversed-phase high-performance liquid chromatography (RP-HPLC), and a colorless oil was obtained. The molecular formula of compound 1 was determined by inspection of the carbon-13 nuclear magnetic resonance (^13^C-NMR) spectrum coupled with high-resolution mass spectrometry (HRMS) data. The 1D and 2D NMR analysis was used to establish its planar structure and relative stereochemistry. However, the absolute stereochemistry of (+)-neopeltolide (**1**) was not determined by Wright and co-workers because of the lack of available material. Then, the absolute stereochemistry of **1** was stablished after the first total synthesis of **1** reported independently by the groups of Panek [13] and Scheidt [14] in 2007 and 2008, respectively. The total synthesis of (+)-neopeltolide (**1**) was reported recently, in 2022, by Fuwa et al. [15]. Neopeltolide (**1**) is an inhibitor of the in vitro proliferation of the A-549 adenocarcinoma of the human lung (IC_50_ = 1.2 nM), the NCI-ADR-RES human ovary sarcoma (IC_50_ = 5.1 nM), and the P388 murine leukemia cell lines (IC_50_ = 0.56 nM). It can also inhibit the growth of the pathogen *Candida albicans* (MIC = 0.62 μg/mL).

Tedanolide (**2**) (Figure 3) is a very potent cytotoxic 18-membered macrolide that was extracted and isolated from the *Tedania ignis*, a very well-known Caribbean marine sponge [16]. *Tedania ignis* samples were collected at Summerland Key, FL, and frozen for transportation [17]. First, the samples were soaked and combined, and then the concentrated extracts were partitioned between hexane and 10% aqueous methanol. Next, the alcoholic layer was diluted to 30% water and extracted with chloroform (CHCl_3_), and the chloroform was chromatographed over Sephadex LH-20. Posterior purification involved chromatography over deactivated silica gel, HPLC, and RP-HPLC. Later, the products of these processes were recrystallized, yielding product **2** as white crystals. Finally, the chemical formula and structure were elucidated using high-resolution fast atom bombardment mass spectroscopy (*HRFABMS*), infrared (IR), NMR spectroscopy, and X-ray diffraction. Tedanolide (**2**) exhibited cytotoxicity against the KB cell line (ED_50_ = 2.5 × 10^−4^ ng/mL) and PS cell line (ED_50_ = 1.6 × 10^−5^ ng/mL) [16] and has demonstrated strong antitumoral activities since it has been found to increase the life span of mice implanted with lymphocytic leukemia by 23% [18].

Lasonolide A (**3a**) (Figure 3) is a 20-membered macrolide isolated in 1994 from shallow waters of the British Virgin Islands in the Caribbean from a marine sponge, *Forcepia* sp. [19]. The biomass was extracted with ethanol (EtOH) and solvent partitioned, followed by reversed-phase vacuum flash column chromatography of the dichloromethane (CH_2_Cl_2_) layer and then purified by semipreparative HPLC to obtain pure lasonolide A (**3a**) as a pale orange oil [20]. Particularly, the isolation process of **3a** was achieved by monitoring each fraction for cytotoxicity and inhibition of cell adhesion. After purification, the cytotoxic studies demonstrated a potent cytotoxicity against A-549 human lung carcinoma (IC_50_ = 40 ng/mL) and P388 murine leukemia (IC_50_ = 2 ng/mL) cell lines [20]. Polarimetry, HRFABMS, 1D and 2D NMR, and IR spectroscopy were used to characterize macrolide **3a**. Specifically, regio- and stereochemistry of the hydroxyl groups, the tetrahydropyran rings, and alkenes of **3a** (Figure 3) were elucidated using rotating-frame Overhauser effect spectroscopy (ROESY) and deuterium exchange ^13^C-NMR experiments. In 2012, Zhang et al. reported promising results using **3a** to induce premature chromosome condensation in different cell lines. For example, lymphoma and leukemia cells showed an 85–95% treatment [21].

In 2004, Wright et al. reported five new lasonolide analogs, lasonolide C (**3c**), lasonolide D (**3d**), lasonolide E (**3e**), lasonolide F (**3f**), and lasonolide G (**3g**) (Figure 3), isolated from the sponge *Forcepia* sp. obtained from the Gulf of Mexico, approximately 100 nautical miles west of Naples, Florida [22]. For the extraction, the sponge was cut into pieces and then extracted with EtOH, followed by a mixture of ethyl acetate and water. The concentrated extract was purified through vacuum flash chromatography with different solvents, and the resulting final fractions were further purified through RP-HPLC. The structures of lasonolides **3c**–**g** were determined employing analytical techniques such as HRFABMS and characterized by spectroscopy (IR, ^1^H-NMR, ^13^C-NMR, correlated spectroscopy (COSY), distortionless enhancement by polarization transfer (DEPT-90 and DEPT-135), heteronuclear multiple quantum coherence (HMQC), and heteronuclear multiple-bond correlation (HMBC)). The main structural difference between lasonolides **3a** to **3g** is the ester derivative lateral chain at C_28_ (Figure 3). At the same time, the lasonolide core remains the same for most of them, except lasonolide G (**3g**), which has an additional long-chain ester at C_20_ (Figure 3). Lasonolides **3c** to **3f** were analyzed for their in vitro proliferation of cancer cells, such as 549 human lung adenocarcinoma (A459), PANC-1 human pancreatic cancer, and NCI-ADR-RES tumor cell lines. Compounds **3c** to **3e** inhibited A559 cells with an IC_50_ of 0.13, 4.5, and 0.31 μM, respectively. For PANC-1 cells, compounds **3c** to **3f** showed inhibition with an IC_50_ of 0.38, 4.89, 0.57, and 15.6 μM, respectively. Only compound **3c** showed good inhibition for the NCIADR-RES cell line with an IC_50_ = 1.12 μM. Lasonolide A (**3a**) was the most biologically active in terms of cytotoxicity against the A459, PANC-1, and NCIADR-RES tumor cells with an IC_50_ of 0.0086, 0.089, and 0.49 μM, respectively.

Looekeyolides A (**4a**) and B (**4b**) are 20-membered macrolides (Figure 3) isolated in 2019 from the lipophilic extracts of black band disease (BBD), a polymicrobial disease consortium dominated by the cyanobacterium *Roseofilum reptotaenium* collected from the Looe Key, Florida, Belize, and Honduras [23]. The samples were used to isolate a unicyanobacterial enrichment culture of *Roseofilum reptotaenium* that was grown and identified by polymerase chain reaction (PCR). The freeze-dried BBD layer was extracted with organic solvents, and the production of secondary metabolites of the lipophilic extracts was analyzed by *low-resolution electrospray ionization liquid chromatography mass spectra* (LRESI-LC-MS). The samples were separated on an RP-HPLC column with a gradient eluent, then fractionated by reversed-phase C_18_ chromatography, followed by reversed-phase C_18_ HPLC to give looekeyolides A (**4a**) and B (**4b**) as major metabolites in the EtOAc partitions by liquid chromatography–mass spectrometry (LC–MS) and ^1^H-NMR analyses. The relatively isolated yields of **4a** and **4b** from different locations and batches highly depend on the extraction and purification conditions. Looekeyolide B (**4b**) is the more stable and oxidized product of looekeyolide A (**4a**). The molecular formula of compounds **4a** and **4b** was confirmed by high-resolution electrospray ionization/atmospheric pressure chemical ionization mass spectrometry (HRESI/APCIMS). The structural analysis of **4a** and **4b** was performed employing spectroscopic methods such as IR, ^1^H- and ^13^C-NMR, double quantum filtered correlated spectroscopy (DQF-COSY), heteronuclear single quantum coherence spectroscopy (HSQC), and HMBC. X-ray crystallography studies established the relative stereochemistry of looekeyolide A (**4a**). As recently as 2020, the analogs looekeyolide C (**4c**) and D (**4d**) (Figure 3) were isolated and characterized from the coral *Siderastrea sidereal*-associated BBD cyanobacterial mats, located in Belize and Florida, and collected between 2014 and 2018 [24]. The extraction and isolation of **4c** and **4d** were performed using the previously reported procedure [23]. Traces of looekeyolide C (**4c**) were detected by low-resolution electrospray ionization mass spectrometry (LRESIMS) but were not isolated for other spectral studies. Nevertheless, the stable looekeyolide D (**4d**) isolation was achieved successfully, obtained as a powder, and characterized by polarimetry and extensive 1D and 2D NMR studies.

Lobophorolide (**5**) (Figure 3) was isolated from the Caribbean brown algae *Lobophora variegate (Dictyotaceae, Phaeophyta)* in the Bahamas, by Kubanek et al. in 2003 [25]. The isolation of 5 was achieved by lyophilization, four times extraction, reduction under vacuum, and subjection to solvent partitioning. The resulting chloroform extract was the only one that possessed all of the antifungal activity and was fractionated using reversed-phase liquid chromatography (LC) in vacuum and silica gel (C_18_). The bioactive fractions obtained were then fractionated and purified twice by size exclusion chromatography with Sephadex LH-20. Finally, compound **5** was obtained as a white amorphous solid after purification by HPLC, first using RP-HPLC and then multiple rounds of normal-phase HPLC with a gradient eluent. The structure elucidation of lobophorolide (**5**) was performed using polarimetry, MS, IR, ^1^H, ^13^C, and 2D NMR spectral analysis, which consists of a 20-membered macrolactone that has attached an aliphatic side chain with a tetrahydropyran ring-termini (Figure 3). Lobophorolide (**5**) displays antifungal activity against *D. salina* and *L. thalassiae* and is highly cytotoxic toward the colon tumor cell line HCT-116 (IC_50_ = 0.03 μ/mL) [26].

In 1994, Shimizu et al. reported the structure of cytotoxic 26-membered macrolide amphidinolide B_1_ (**6a**) (Figure 3) and isomers isolated for the marine dinoflagellate *Amphidinium* sp., located in St. Thomas, U.S. Virgin Islands [27]. Interestingly, Ishibashi et al. in 1987 reported, for the first time, the isolation and structure determination of amphidinolide B_1_ (**6a**) from the Okinawan flatworm *Amphiscolops* sp. [28]. Later, in 1989, was reported the structure revision of **6a** [29] and, finally, in 1994, was reported the absolute stereochemistry by synthesis of a degradations product [30]. The freeze-dried algal cells were extracted, and the solvent was partitioned between *n*-hexane and 90% methanol (MeOH). The methanol layer was separated successively on silica gel, C_18_ silica gel, and Hamilton PRP-1 and CN silica gels to afford pure compounds (**6a**–**c**) (Figure 3). Shimizu and coworkers confirmed that the 1D and 2D NMR spectra of amphidinolide B_1_ (**6a**) were comparable with previous reports in the literature [26,29]. The relative stereochemistry of amphidinolide B_1_ (**6a**) was established by X-ray analysis, confirming that this macrolide has a rectangular shape and an internal hydrogen bond bridge between the C_26_-OH group and the oxygen atom of the epoxide (Figure 3). The amphidinolide isomers B_2_ (**6b**) and B_3_ (**6c**) were confirmed based on the NMR analysis of the coupling constants, revealing that B_2_ (**6b**) and B_3_ (**6c**) are the C_18_ and C_20_ epimers, respectively, to B_1_ (**6a**) (Figure 3). Amphidinolides B_1_ (**6a**) (IC_50_ = 0.120 μg/mL), B_2_ (**6b**) (IC_50_ = 7.5 μg/mL), and B_3_ (**6c**) (IC_50_ = 0.206 μg/mL) exhibited potent cytotoxicity against the human colon tumor cell line HCT 116. Amphidinolide B_1_ (**6a**) showed lower cytotoxicity against the murine leukemia cell line L1210 (IC_50_ = 1.4 × 10^−4^ μg/mL) [28].

In 1995, caribenolide I (**7**) (Figure 3), a polyketide 26-membered macrolide (macrocyclic lactone), was reported and extracted from a single-cell, free-swimming dinoflagellate organism known as *Amphidinium* sp., found and collected from St. Thomas, U.S. Virgin Island [31]. The dried cells were mixed with solvent, then sonicated and filtered with Celite until the filtrate turned pale green. Next, the extracts were concentrated, partitioned, and re-extracted. The resulting extract was purified by column chromatography, and compound **7** was eluted in the first mixture of solvents and amphidinolide B_1_ (**6a**). After further purification using reversed-phase chromatography on a C_18_ silica gel and later with HPLC, pure caribenolide I (**7**) was obtained. The structure of **7** contains a macrocyclic lactone with 13 stereogenic centers, an α-methylene epoxide, and a furan ring confirmed by NMR analysis, although the absolute and relative stereochemistry is still under investigation (Figure 3). Other groups have investigated the enantioselective partial or total synthesis of **7** to determine the relative and absolute configuration for later comparison with the natural product [32,33,34]. Caribenolide I (**7**) showed potent in vitro cytotoxic activity against both the human colon tumor cell line HCT 116, and its drug-resistant cell line, HCT 116/VM 46 (IC_50_/HCT116/WT = 0.001 µg/mL or 1.6 nM), indicating that it has antitumor characteristics. This cytotoxicity is 100 times higher than that observed for amphidinolide B_1_ (**6a**) (IC_50_/HCT116/WT = 0.120 µM). Comparing these biological activities, caribenolide I (**7**) is more potent than amphidinolide B_1_ (**6a**).

Three 26-membered macrolides, bryostatins 16 (**8a**), 17 (**8b**), and 18 (**8c**) (Figure 3), were isolated from the marine bryozoan *Bugula nertina* L. which was recollected from the Gulf of Mexico in Florida [35]. Assays against the murine P388 lymphocytic leukemia cell line guided the separation. To isolate bryostatin 16–18 (**8a**–**c**), a column chromatography separation with Sephadex LH-20 and silica gel was utilized. One of the fractions was separated by HPLC on silica gel containing bryostatin 16 (**8a**) and bryostatin 17 (**8b**). After normal phase silica gel semipreparative HPLC, bryostatin 18 (**8c**) was obtained. Specifically, the growth inhibitory activity against murine P388 lymphocytic leukemia for bryostatins 16 (**8a**), 17 (**8b**), and 18 (**8c**) showed an ED_50_ of 9.3 × 10^−3^ μg/mL, 1.9 × 10^−2^ μg/mL, and 3.3 × 10^−3^ μg/mL, respectively. Compounds (**8a**–**c**) showed the greatest activity against the lung (NCI-H460) cell line. Bryostatin 16 (**8a**) showed inhibitory activity against ovarian (OVCAR-3: GI_50_ = 1.9 μg/mL), CNS (SF-295: GI_50_ = 1.4 μg/mL), renal (A498: GI_50_ = 1.9 μg/mL), lung (NCI-H460: GI_50_ = 0.019 μg/mL), colon (KM20L2: GI_50_ = 2.2 μg/mL), and melanoma (SK-MEL-5: GI_50_ = 1.2 μg/mL). The results were similar for the bryostatins 17 (**8b**) and 18 (**8c**) against the same mini-panel. The MS analysis reveals that bryostatins 16 (**8a**) and 17 (**8b**) have the same molecular formula, and ^1^H- and ^13^C-NMR spectral analyses were employed to assign the structures, and the 2D NMR spectral data confirmed that they are *E/Z* stereoisomers at the C_21_ to C_34_ atoms (Figure 3). In contrast, bryostatin 18 (**8c**) has a different molecular formula (C_42_H_64_O_15_), obtained from the HRFABMS data. The comparison of the NMR spectra analysis of bryostatin 18 (**8c**) with previously reported bryostatin 10 (**8d**) demonstrated that they are geometrical isomers [36]. Prorocentrolide B (**9**) (Figure 3) is a toxin that was isolated in 1996 from the tropical dinoflagellate, *Prorocentrum Maculosum Faus* that was obtained from Dauphin Island, Alabama [37]. Prorocentrolide B (**9**) is classified as a 28-membered macrocyclic lactone and a 26-membered carbo-macrocycle, a cyclic imine group, and cyclic ether (Figure 3). To isolate macrolide **9** from *P. maculosum*, cells were sequentially extracted, solvent partitioned, and extracted again, and the bioassays of the fractions indicated where the toxins were present and confirmed by thin-layer chromatography (TLC). The toxins were purified by gel permeation (Sephadex LH-20 open column). The toxin was chromatographed on a reverse phase and HPLC to isolate prorocentrolide B (**9**). Macrolide **9** is a fast-acting toxin but is not a phosphate inhibitor. However, because it contains a cyclic imine, this suggests that its functionality must be significant in biological activities, but it was not determined [37]. The structural elucidation of **9** was carried out by ^1^H and ^13^C NMR analysis, confirming that it has seven rings, and 2D NMR analysis was employed to determine the relative structure of the six- and five-membered cyclic systems (Figure 3). Unfortunately, the absolute stereochemistry of **9** remains unsolved.

**Figure 4:**
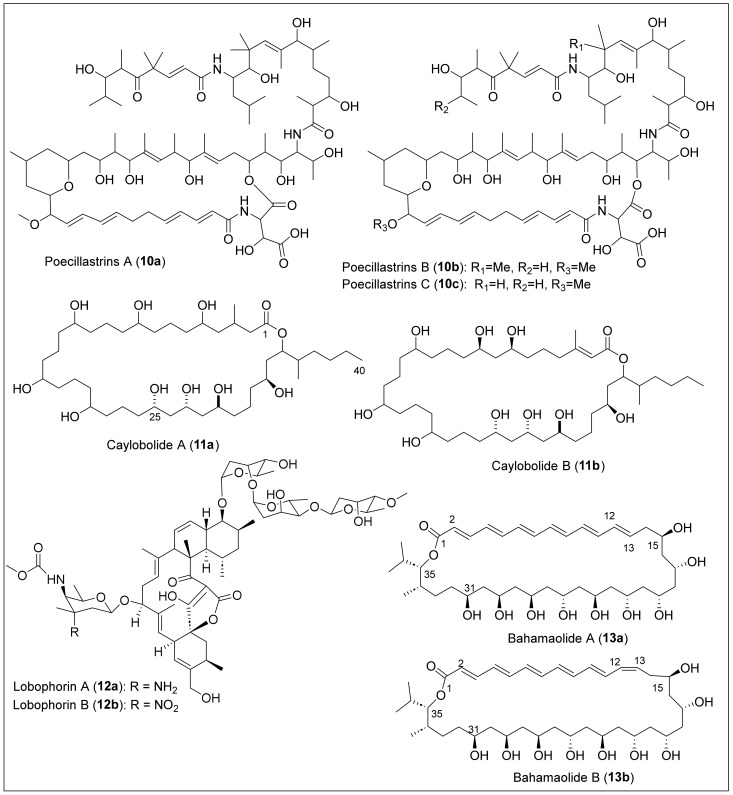
Structures of Caribbean macrolides **10**–**13**.

In 2002, the isolation and structural elucidation of poecillastrin A (**10a**) (Figure 4) was reported. This 33-membered macrocyclic lactam was extracted from the marine sponge *Poecillastra* sp., obtained from Grand Bahama Island, Bahamas [38]. Poecillastrin A (**10a**) was purified after a multistep fractionation of the *Poecillastra* sp. extract, and purification by HPLC, HRFAB, and MS measurement established the molecular formula of **10a**. The structure of **10a** was elucidated after extensive 1D and 2D NMR spectral analysis, but the relative and absolute configuration remains undetermined (Figure 4). The study of **10a** against four different human tumor cell lines and two murine mast cell lines was differentially cytotoxic and antiproliferative, with an EC_50_ that ranged from <25 nM to >10,000 nM. Later, in 2007, two new bioactive 35-membered macrolide lactams, poecillastrins B (**10b**) and C (**10c**) (Figure 4), were isolated and derived from the same sponge *Poecillastra* sp. and the same location as poecillastrin A *(***10a**) [39]. First, to isolate and purify the two analogs, poecillastrins (**10b**–**c**), a partition was realized with the sponge to separate the components in two phases. Next, the crude extract product from the sponge was extracted again using 90% methanol in water and *n*-hexane. Then, the aqueous phase with methanol was dissolved in water and extracted again using chloroform. This process purified the liquid phase with chloroform by gel filtration on Sephadex LH-20 to produce four fractions. The first two fractions were purified again using wide-pore column chromatography and then separated using reversed-phase HPLC. To evaluate the bioactivity of poecillastrin B (**10b**) and C (**10c**), DMSO solutions of the samples were diluted and mixed with human melanoma tumor cell line (LOX) cell cultures to evaluate their effects. They demonstrated promising cytotoxicity results against tumor cells with an IC_50_ value of less than 1 μg/mL.

A 36-membered macrolide, caylobolide A (**11a**) (Figure 4), was reported in 2002 and isolated from the Bahamian cyanobacterium *Lyngbya majuscula*, collected in 1999 in the Bahamas [40]. The caylobolide A (**11a**) macrolactone contains an unprecedented, repeated unit—a contiguous pentad of 1,5 diols- and 1,3,5-triol units and eleven chiral centers (Figure 4). Specifically, the biomass sample was lyophilized, extracted, filtered, and concentrated to give a dark-green extract. A sequential solvent partition of the extract produced a final fraction subjected to Sephadex LH-20 chromatography to give six fractions. Only fraction three was applied to a silica C_18_ cartridge, eluted with a gradient, and subjected to C_18_ HPLC to give **11a** as a white solid. Caylobolide A (**11a**) exhibited in vitro cytotoxicity toward HCT-116 human colon tumor cells with an IC_50_ = 9.9 µM, but showed no significant antifungal activity against *Candida albicans* or *Candida glabrata*. Then, in 2010, the macrolactone caylobolide B (**11b**) was isolated from *Phormidium* sp. samples collected from Key West, Florida [41]. The freeze-dried sample of *P. inundated* was first extracted, solvent partitioned and subjected to bioactivity-guided isolation using silica gel chromatography and RP-HPLC to yield caylobolide B (**11b**) as a colorless amorphous solid (Figure 4). Caylobolide B (**11b**) displayed cytotoxic activity against HT29 colorectal adenocarcinoma (IC_50_ = 4.5 μM) and HeLa cervical carcinoma cells (IC_50_ = 12.2 μM). The structure elucidation of **11a** and **11b** was carried out by MS and NMR experiments, and the relative and absolute configuration of the contiguous 1,5-diols and *syn/anti*-1,3,5-triol subunits at C_25_-C_33_ in caylobolides A (**11a**) and B (**11b**) (Figure 4) was assigned using Kishi’s Universal NMR database and the Mosher ester analysis. The relative configuration of the *syn*-1,3-diol at C_7_-C_9_ in caylobolide B (**11b**) was also determined with Kishi’s Universal NMR Database. However, the synthesis of C_25_–C_40_ and C_21_–C_40_ fragments of **11a** were reported in 2011 [42] and 2014 [43], respectively (Figure 4). The complete absolute configuration of all chiral centers in caylobolides (**11a)** and (**11b)** remains undetermined.

Lobophorins A (**12a**) and B (**12b**) (Figure 4) are macrolides that have attached glycosidic linkages that were isolated from the marine bacterium in the surface of Caribbean brown algae *Lobophora variegate*, collected from Belize [44]. Lobophorin A (**12a)** has a molecular composition of C_61_H_92_N_2_O_9_, while lobophorin B (**12b)** has C_61_H_90_N_2_O_21_. Comparing lobophorins (**12a**) and (**12b**), the only structural difference is the axial R group at the amino sugar, an amino group in **12a,** and a nitro group in **12b** (Figure 4). The saline fermentation broths were filtered and extracted by percolation with C_18_ chromatography resin in isolation. Then, a dark-brown oil was obtained through elution and solvent removal with MeOH. Using flash chromatography with silica gel, the extract was fractioned and purified using RP-HPLC with silica gel. This produced lobophorins A (**12a**) and B (**12b**) as amorphous and polar solids. Lobophorin A (**12a**) was obtained after elution with 75% MeOH with 0.5% NH_4_Ac, while lobophorin (**12b**) was obtained after elution with EtOAc. After assays, lobophorins A (**12a**) and B (**12b**) present potent anti-inflammatory activities and are inhibitors of topical PMA-induced edema in mice ears. In comparison with other related antibiotic macrolides, lobophorins **12a** and **12b** do not present antibiotic properties. Other studies demonstrated that lobophorin A (**12a**) had bioactivity against *Escherichia coli* and lobophorin B (**12b**) against *Staphylococcus aureus* [45].

#### 1.1.2. Polyene Macrolides

Larger macrocyclic compounds (from 26 to 38 atoms) have been found in the subgroup of macrolides called polyene antifungals. Their structure is characterized by a large conjugated polyene chain, all with *E* geometry, and a polyol or a mixture of polyol and polypropionate chains. Polyenes macrolides have been known as antifungal compounds, and the medicinally important ones include amphotericin B [46,47,48] and nystatin [49]. Bahamaolides A (**13a**) and B (**13b**) (Figure 4), antifungal polyene polyol 36-membered macrolides, were isolated from the culture of the marine actinomycete *Streptomyces* sp., derived from a sediment sample collected in the Bahamas [50]. The Caribbean antifungal polyene polyol 36-membered macrocyclic lactones **13a** and **13b** (Figure 4) belong to the hexaene macrolide class, and they have the same molecular formula of C_39_H_64_O_11_, determined by HRFABMS and ^1^H- and ^13^C-NMR data analysis. The structures of **13a** and **13b** were determined by 1D and 2D NMR, MS, and IR spectra analysis, demonstrating that bahamaolides (**13a**–**b**) have a planar structure bearing a hexaene chain (C_2_–C_13_) and nine consecutive *syn/anti* 1,3-diols chain (C_15_–C_31_) (Figure 4). The absolute configurations of **13a** were established by combined multistep reaction synthesis, such as the Mosher method and derivatization with acetals and spectroscopic data (^1^H and ^13^C, COSY, HSQC, HMBC, and ROESY NMR). Compounds **13a** and **13b** are *trans* and *cis* isomers, respectively, because the only difference is the alkene geometry at C_12–13_ (Figure 4). Bahamaolides (**13a**) and (**13b**) were isolated by combining normal-phase and reversed-phase chromatographic techniques. The extract was fractionated by flash column chromatography on silica gel using a step elution with mixtures of organic solvents; bahamaolides (**13a**) and (**13b**) eluted in the ethyl acetate/methanol fraction. Semipreparative reversed-phase HPLC further purified this fraction to obtain pure compounds **13a**–**b** as yellow powders. Bahamaolide A (**13a**) showed moderate antifungal activity against *Aspergillus fumigatus*, *Trichophyton rubrum*, *T. mentagrophytes*, and *Candida albicans*, but bahamaolide B (**13b**) did not display significant inhibitory activity against any tested strains. Bahamaolide A (**13a**) showed significant isocitrate lyase (ICL), and inhibitory activity against *C. albicans*, while bahamaolide B (**13b**) showed weak activity. Bahamaolides (**13a**) and (**13b**) were also tested in antibacterial assays against gram-positive and gram-negative bacteria but did not show significant activity. Finally, the cytotoxicity was measured against several human cancer cell lines, including lung, colon, breast, and liver cancer. However, they did not display significant inhibitory activity, even at high concentrations (100 μg/mL).

### 1.2. Linear Polypropionates

**Figure 5:**
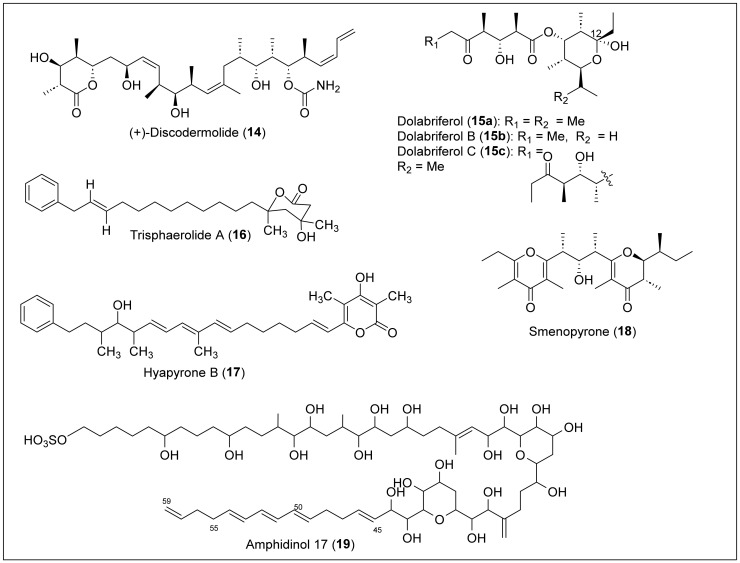
Structures of Caribbean linear polyketides **14**–**19**.

Some organisms, such as marine mollusks, microorganisms, terrestrial plants, and insects, produce a rare class of acyclic secondary metabolites that can be classified as linear polypropionates, such as mupirocin, which is used clinically as an antibiotic [8,9]. (+)-Discodermolide (**14**) is a linear polypropionate that contains a six-membered lactone that was isolated for the first time by Gunasekera et al. in 1990 from the marine sponge *Discodermia dissolute*, located in Grand Bahama Island (Figure 5) [51]. Then, in 2013, Ruiz et al. reported the isolation from Santa Marta, Colombia [52]. The solvent extraction and partition, followed by silica gel chromatography and RP-HPLC, produced **14** as a crystalline solid. Structural determination and absolute configuration of **14** were performed by FABMS, NMR, IR, and X-ray analysis [51]. The structure of **14** has an amide, a polypropionate segment with 13 chiral centers (Figure 5). Discodermolide (**14**) exhibited strong cytotoxicity against murine P388 leukemia (IC_50_ = 0.5 μg/mL), murine splenocytes (IC_50_ = 0.5 μg/mL), and human peripheral (IC_50_ = 5 μg/mL) blood lymphocytes [9]. This natural product has been approved for clinical trials regarding antitumoral activity and has also shown strong antimitotic, antifungal, and immunosuppressant activity [53]. However, further research is required to approve this product as a chemotherapeutic, seeing that the product presented pneumotoxicity in the preliminary clinical trial results.

Gavagnin and co-workers isolated dolabriferol (**15a**–**c**) from the anaspidea mollusk *Dolabrifera dolabrifera*, collected from Cuba in 1990 [54]. The biomass was dissected and extracted with acetone, analyzed by TLC, and then soaked in acetone using ultrasound treatment. The acetone extract was submitted to a preparative chromatographic column, and further purification using a silica column yielded pure dolabriferol (**15a**). The HREIMS analysis determined the molecular formula of **15a** (C_21_H_38_O_6_) (Figure 5). NMR methods and X-ray analysis determined the relative and absolute stereochemistry of the **15a** structure. Dolabriferol (**15a**) consists of two polypropionate subunits linked by an ester in which one of the polypropionate chains has a hemiacetal at C_12_. The related compounds, dolabriferols B (**15b**) and C (**15c**), were isolated by A. Rodríguez et al. from a Caribbean mollusk, *Dolabrifera dolabrifera*, that was collected in Puerto Rico. The organism was lyophilized, extracted, filtered, and concentrated to yield a dark green oil. The sample was later suspended in H_2_O and extracted with organic solvents, concentrated, and separated in fractions with silica gel chromatography to yield dolabriferol (**15a**), dolabriferol B (**15b**), and dolabriferol C (**15c**) (Figure 5). Dolabriferols (**15a**–**c**) were characterized using spectral data and X-ray analysis [55]. Dolabriferol (**15a**) did not display any significant activity, but dolabriferols B (**15b**) and C (**15c**) showed inhibitory activity (39% and 93%, respectively) against *Mycobacterium tuberculosis* H37Rv at a concentration of 128 μg/mL.

Trisphaerolide A (**16**) (Figure 5) was isolated from the purple sponge *Erylus trisphaerus*, collected in Dominica in 2000 [56]. This natural product **16** can be classified as a linear polyketide with a phenyl terminus and a 6-membered propionate-derived lactone. It was isolated by extracting with methanol, later passed by a lipophilic gel column, concentrated, and then separated by gel permeation chromatography (Sephadex LH20) and silica gel centrifugal chromatography. Trisphaerolide A (**16**) was purified with normal-phase HPLC using a mixture of ethyl acetate and hexane as the mobile phase. The structural characterization and the relative configuration of **16** (Figure 5) were performed by HREIMS, ^1^H, ^13^C, DEPT, COSY, HSQC, and HMBC NMR data. Trisphaerolide A (**16**) does not inhibit the G_2_ cell cycle checkpoint but has a mild toxicity inhibition towards breast cancer cells with an IC_50_ of 30 µg/mL.

Okanya and co-workers reported the isolation and purification of hyapyrone B (**17**) (Figure 5) from the bacterial *Hyalangium minutum* strain Hym-3 [57]. This strain was from a soil sample collected in 1997 in the Saint Vincent and the Grenadines Islands within the Windward Islands. After culturing the bacterial strain, an extract was eluted with methanol/acetate. After two partitions, the extract was separated by silica gel flash chromatography. Finally, the sample was run through RP-HPLC for further purification of hyapyrone B. High-resolution electrospray ionization mass spectroscopy (HRESIMS) and NMR analysis were performed to determine that the molecular formula of **17** was C_33_H_44_O_4_. Based on the structure, we can classify **17** as a 2-pyrone polypropionate compound containing a phenyl terminal like compound **16**, a short propionate, and conjugated triene chains (Figure 5). The best result for antibiotic activity of **17** was against the gram-positive bacterium *N. flava*, with a MIC value of 33 μg/mL. In addition, hyapyrone B (**17**) showed cytotoxic activity against the mouse fibroblast cell line L929, with a 20 μM IC_50_ value. It also had the best antifungal activity compared with others in its family, and showed a MIC value of 33 μg/mL against *M. hiemalis*. However, the authors say these results are small and do not represent significant biological activity for **17**.

Smenopyrone (**18**) (Figure 5) is a 4-pyrone polypropionate secondary metabolite isolated from a marine sponge, *Smenospongia aurea*, located in the Bahamas Islands [58]. For the isolation, the sample was cut into small pieces, homogenized, extracted, partitioned between the organic and aqueous layers, separated, and filtered. The organic extract was chromatographed, partitioned, and subjected to repeated RP-HPLC separations to afford pure smenopyrone (**18**). The structural determination of **18** was performed by MS and NMR (1D and 2D) analysis. The absolute configuration of **18** was based on its electronic circular dichroism (ECD) spectrum, and the pyrone polypropionate structure has a γ-dihydropyrone and a γ-pyrone (Figure 5). Pyrones have recently been recognized as bacterial signaling molecules, so it is speculated that smenopyrone (**18**) could mediate inter-kingdom chemical communication between *S. aurea* and its symbiotic bacteria. It is noteworthy that no biological activity studies have been reported in the literature.

Amphidinol 17 (**19**) (Figure 5) was isolated from a Bahamas strain of an *Amphidinium carterae* sample collected in Little San Salvador Island, Bahamas, in 2004 [59]. *A. caterae* cells were extracted, concentrated, partitioned, and then fractionated using a Sep-Pak C_18_ column using a stepped gradient. After LC-MS analysis and HPLC purification, compound **19** was obtained. High-resolution mass spectrometry (HRMS) was used to confirm that the molecular formula of compound **19** was C_63_H_110_O_24_S, and ultraviolet (UV) spectroscopy confirmed the presence of a conjugated triene chromophore at C_45_-C_59_. The NMR data analysis with COSY, total correlation spectroscopy (TOCSY), HSQC, and HMBC spectra were used to assign the structure of **19** (Figure 5). However, the relative and absolute configuration was not determined. The structure of **19** contains long polyol and polyene chains, a sulfonate group at the end of the chain, and two cyclic esters. Amphidinol 17 (**19**) showed potent hemolytic activity against human red blood cells (EC_50_ = 4.9 μM) and no detectable antifungal activity against the fungi *Aspergillus niger* and *Candida kefyr*.

### 1.3. Cyclic Polyethers

**Figure 6:**
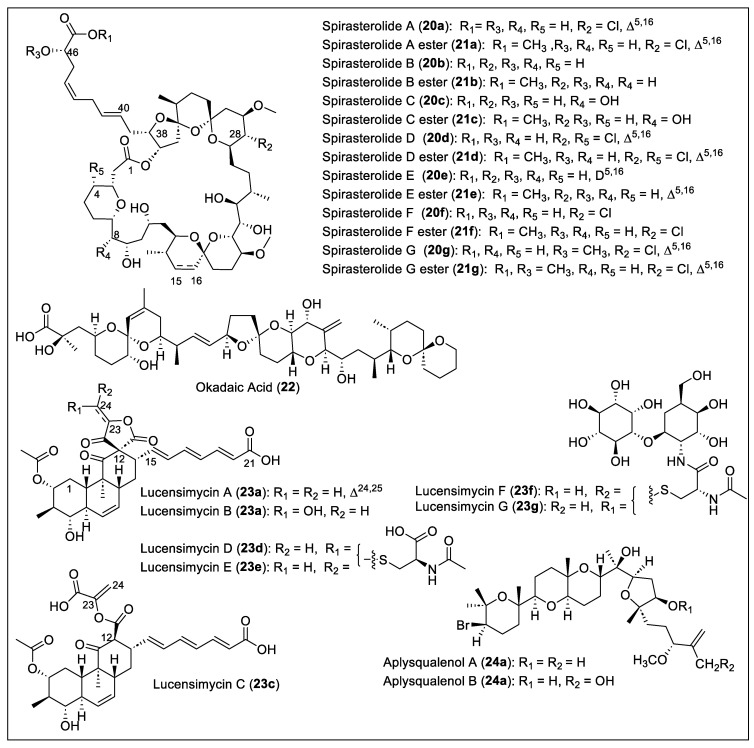
Structures of Caribbean polycyclic ethers **20**–**24**.

Cyclic polyether antibiotics are a large group of secondary polypropionate metabolites that show bioactivities such as antibacterial, antifungal, antiparasitic, antiviral, and tumor cell cytotoxicity [60]. In addition, several tetrahydrofurans characterize them; tetrahydropyran fused rings and spiroketals. The 38-*membered* macrolactones spirastrellolide A (**20a**) [61,62], spirastrellolide B (**20b**) [63], and spirastrellolides C to G (**20c–g**) [64] (Figure 6) were extracted and isolated from the Caribbean sponge, *Spirastrella coccinea*, collected at Capucin, Dominica, by R. Andersen and coworkers. Spirastrellolide A (**20a**) was first reported, then spirastrellolide B (**20b**), and finally spirastrellolides C (**20c**), D (**20d**), E (**20e**), F (**20f**), and G (**20g**) (Figure 6). For the extraction and isolation of spirastrellolide A (**20a**), a sample of the sponge was extracted exhaustively with MeOH, solvent partitioned, and purified by column chromatography to obtain **20a** and then converted in the **21a** methyl ester (R_1_ = Me) after reaction with diazomethane (Figure 6). Normal and reversed-phase flash chromatography was performed, then RP-HPLC to obtain the pure ester **21a** as an oil. To characterize and determine the structure of compounds **20a** and **21a**, MS and NMR spectral data (HSQC, chemical ionization high-resolution mass spectrometry (CI-HRMS), COSY, HMBC, NOESY, and ROESY) were analyzed. In 2004, the Anderson group reported the revised structure of **20a** based on the new MS data, chemical transformations such as acetonides, acetylation, deuterium exchange, and ROESY correlations a *J* coupling constants [62]. In 2007, the identification of the structure of spirastrellolide B (**20b**) and the corresponding methyl ester **20b** were reported, which were elucidated and analyzed by HRESIMS and NMR spectroscopy [63]. The structural differences between spirastrellolides A (**20a**) and B (**20b**) are the presence of the chlorine substituent at C_28_ and an unsaturation (Δ^15,16^) in spirastrellolide A (**20a**), while, in spirastrellolide B (**20b**)**,** both groups were missing (Figure 6). Spirastrellolide B (**20b**) was chemically transformed into a derivative by oxidative cleavage of the side chain olefins and converted into a C_40_ ester that was analyzed by X-ray crystallography to determine the absolute configuration of the spirastrellolides macrolide core [63]. The remaining five new spirastrellolides C to G (**20c–g**) were isolated and extracted after their conversion into the corresponding C_47_ methyl esters (**21c–g**) following the reported procedure (Figure 6) [64]. The detailed characterization of the methylspirastrellolides (**21c–g**) was carried out by MS (HRESIMS and LRESIMS) analysis and 2D NMR (HSQC, HSQC-TOCSY, COSY, TOCSY, ROESY, and HMBC) data. The most important achievement during the structural analysis of **20c–g** was that the determination of the absolute configuration at the C_46_ of spirastrellolides (**20a–g**) was R (Figure 6). Spirastrellolides (**20a–g**) have a unique chemical structure with 21 stereogenic centers, tetrahydropyran, several spiroketals embedded in the macrocycle, and a side chain terminating in a carboxylic acid (Figure 6). Methylspirastrellolide A (**21a**), at an IC_50_ of 100 ng/mL, exhibits potent activity in a cell-antimitosis (antimitotic) assay [61]. Spirastrellolide A (**20a**) has led to the understanding of diseases such as Parkinson’s and Alzheimer’s disease due to the selective inhibition of protein phosphatases: PP2A potently (IC_50_ = 1 nM), PP1 much less potently (IC_50_ = 50 nM), and PP2C not at all [62]. The cell-based assay for premature mitosis for methylspirastrellolides A (**21a**) and C (**21c**) showed the same IC_50_ value of 0.4 μM, and both methylspirastrellolides D (**21d**) and E (**21e**) showed an IC_50_ of 0.7 μM.

The isolation of okadaic acid (**22**) (Figure 6) from *Halichondria melanodocia*, a Caribbean sponge collected in the Florida Keys (Summer key), was reported in 1981 by Tachibanat and Scheuer [65]. The extract of the sponge *H. melanodocia* was extracted continuously and consecutively with CH_2_Cl_2_, 10% aqueous MeOH, hexane, CCl_4_, and CHCl_3_. The resulting organic extract was chromatographed over Sephadex LH-20. Only the fractions that showed mouse toxicity were chromatographed over deactivated silica gel to give the toxic component **22** as a brown powder. A white crystalline solid of **22** was obtained after crystallization from benzene. Compound **22** exhibited toxicity against P388 (ED_50_ = 1.7 × 10^−3^ mg/kg) and L 1210 cell lines (ED_50_ = 1.7 × 10^−2^ mg/kg), and, when subtoxic doses were tested in vivo against P388 lymphocytic leukemia, no activity was found. The ^1^H- and ^13^C- NMR spectra, IR, UV, and MS analyses were employed to determine the absolute configuration of **22** (Figure 6). The isolation of okadaic acid (**22**) was reported in 1990 from the Caribbean dinoflagellate *Prorocentrum concavum*, a source of the toxins related to ciguatera fish poisoning. A strain of the species *P. concavum* was isolated from epiphytic macroalgae at Salt Island in the British Virgin Islands [66]. The culture was filtrated, sedimented, and centrifuged. All cells were extracted with methanol and partitioned. The supernatants were combined and then extracted using chloroform. These extracts were then chromatographed on silica gel with chloroform–methanol gradients. Toxic fractions were separated by C_18_ RP-HPLC from silica gel and then were assessed by mouse bioassay or by antimicrobial activity against *Candida albicans*. The most poisonous fraction (LD_50_ = 210 ± 15 μg/kg, i.p. in mice) afforded **22** as a colorless crystalline material from a methanol solution. Okadaic acid (**22**) was identified by ^1^H-NMR analysis and compared with the previously reported NMR spectra [65].

Lucensimycin A (**23a**) and B (**23b**) (Figure 6) were isolated and extracted by Singh et al. from the bacterial strain *Streptomyces lucensis* MA7349 from a soil sample collected in Martinique Island [67]. After fermentation, the bacteria were extracted with ethyl acetate, concentrated, dissolved in MeOH-H_2_O, and washed with hexanes. Then, the organic layer was purified by chromatography (Sephadex LH20) and RP-HLPC to produce **23a** as a significant component and **23b** as the minor metabolite. Compounds **23a** and **23b** were characterized by mass spectral analysis, UV, and IR spectrum, ^1^H and ^13^C-NMR data comparison, and 2D NMR correlations (COSY, HMQC, NOESY, NOE, and DEPT). The **23a** and **23b** structures have three trans-fused cyclic systems, a unique five-membered spiro-γ-lactone, and all trans-trienoic acid chains (Figure 6). The absolute configuration of **23a** and **23b** was also determined. Compound **23b** differs from **23a** at the C_23_–C_24_ because the terminal olefin is hydrated, forming the primary alcohol at C_24_ (Figure 6). Later, in 2008, was reported the discovery of lucensimycin C (**23c**) (Figure 6) from *S. lucensis* MA7349 [68]. The fermentation broth was extracted with acetone and separated by reversed-phase column chromatography (twice) to obtain lucensimycins A (**23a**) and C (**23c**). The physical and spectral data allowed us to characterize the new compound **23c**, which does not have the five-membered bis-lactone at C_12_ but has an open acid version (Figure 6). The absolute stereochemistry of **23c** was determined by Mosher ester and acetylation methodologies. Lucensimycins A (**23a**) and B (**23b**) at 10 μg show a large zone of inhibition of 14 mm and 9.3–6.8 mm, respectively, against the antisense rpsD strain compared with the control *S. aureus* (12.1 mm) [67]. Lucensimycin C (**23c**) was 80-fold less active than **23a** because it showed an 8 mm clearance zone at 100 μg against *S. aureus* [68]. No significant activity (MIC = 250 μg/mL) was shown by compounds **23a–c** against wild-type *S. aureus*. A year later (2009), Singh and coworkers reported the isolation of four new compounds, lucensimycins **D** to **G** (**23d–g**) (Figure 6), from the location and strain of *S. lucensis* MA7349 [69]. To extract and isolate these new compounds, **D–G** (**23d–g**), the previously reported procedure was employed [68]. MS, UV, IR, and NMR spectral analysis were used to characterize all of them. Lucensimycins D (**23d**) and E (**23e**) are isomers with an *N*-acetyl cysteine unit at C_24_ (Figure 6). Lucensimycin isomers F (**24f**) and G (**23g**) have *N*-acetyl cysteine linked with a glycosidic linkage with *myo*-inositol and 2-amino-2-deoxy-L-idose (Figure 6). The biological activity assays against *S. aureus rps*D antisense showed 12 mm of the zone of inhibition for **23d** and **23e** at 10 μg, which are the most active and selective of the lucensimycins series. Lucensimycins **23f** and **23g** were not assayed against *S. aureus rps*D antisense. Lucensimycin E (**23e**) showed the most significant activity for inhibition of the *S. aureus* Smith strain (MIC = 32 μg/mL) and *Streptococcus pneumoniae* CL 2883 (MIC = 8 μg/mL). Lucensimycin D (**23d**) showed only inhibition of *S. pneumoniae* (MIC = 250 μg/mL) and was not active against *S. aureus*. The other lucensimycins (**23e–f**) at 250 μg/mL did not have any antimicrobial activity against other gram-positive and gram-negative bacteria, and *Candida albicans*. Due to the weak biological activity of lucensimycins (**23a–g**), the authors decided not to continue further studies.

Both aplysqualenols A (**24a**) and B (**24b**) (Figure 6) originate from the Caribbean sea slug *Aplysia dactylomela*, which was collected from Puerto Rico by Rodríguez et al. [70]. They can be classified as brominated polycyclic ethers. To isolate and purify **24a** and **24b**, an extract of freeze-dried *A. dactylomela* was extracted with MeOH/CHCl_3_ (1:1). The organic extracts were concentrated, and the residue was suspended in water. It was partitioned between CHCl_3_, butanol, EtOAc, and hexane. This was then subjected to column chromatography with silica gel and CHCl_3_ in hexane. Many fractions were made and purified on silica gel. NP-HPLC and CC on silica gel with EtOAc in hexane, and hexane/2-propanol (95:5) were used to obtain pure aplysqualenol A (**24a**). The portion eluting with EtOAc/MeOH (8:2) was purified on a Bio-Beads SX-3 column and given various fractions. One of these fractions was chromatographed with silica gel and MeOH in CHCl_3_ to obtain pure aplysqualenol B (**24b**). Aplysqualenol A (**24a**) has inhibitory activity against SNB-19 CNS cancer (IC_50_ = 0.4 μg/mL) and T-47D breast cancer lines (IC_50_ = 0.4 μg/mL). In addition, aplysqualenol A (**24a**) showed 90% of maximum response (EC_90_) of antiviral activity at concentrations above 4 μg/mL, observed against various viruses such as herpes simplex virus type 1 (HSV-1) and type 2 (HSV-2) varicella zoster virus (VZV) and human cytomegalovirus (HCMV). Compound **24a** showed high toxicity (EC_90_ = 0.08 μg/mL) against the Epstein–Barr virus (EBV) and showed no inhibitory activity for in vitro studies against *Mycobacterium tuberculosis* H37Rv. Moderate activity against *Plasmodium falciparum* was observed with aplysqualenol A (**24a**) (IC_50_ = 11 μg/mL) and B (**24b**) IC_50_ = 18 μg/mL).

**Figure 7:**
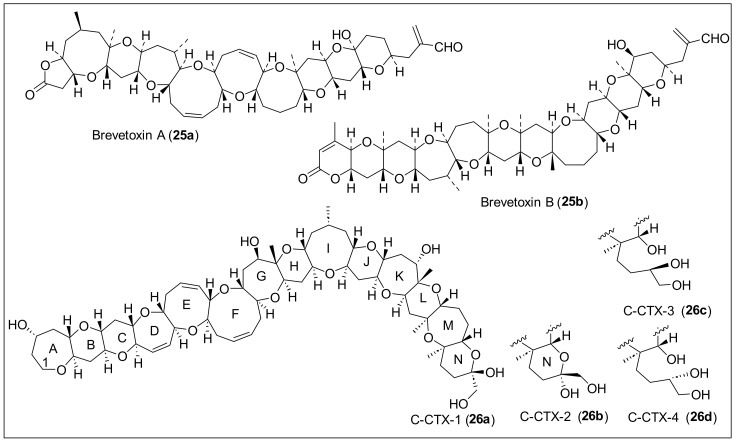
Structures of Caribbean polycyclic ethers **25** and **26**.

Brevetoxins (BTX) A (**25a**) and B (**25b**) (Figure 7) were isolated from the ‘red tide’ dinoflagellates *Ptychodiscus brevis* that were formerly known as *Gymnodimium breve* [71,72]. These *P. brevis* toxins are associated with ‘red tide’ blooms in Florida and the Gulf of Mexico. The natural product groups of brevetoxins are classified as complex polycyclic ethers. BTXA (**25a**) was isolated from the cultured cell of *G. brevis* by solvent partition and successive chromatographic purifications [71]. After acidification to pH 5.5, a crude mixture of brevetoxins was obtained after extraction with diethyl ether, and repeated flash chromatography produced the BTXA (**25a**), BTXB (**25b**), and BTXC (the structure is not shown) congeners (Figure 7) [72]. Brevetoxins A (**25a**) and B (**25b**) have molecular formulas of C_49_H_70_O_13_ and C_50_H_70_O_14_, respectively, determined by MS. The IR and NMR spectral analysis confirms that BTXA (**25a**) has ten rings. In contrast, BTXB (**25a**) has 11 rings containing both compounds, *trans*-used cyclic ethers, varying in size from six to eight members (Figure 7). Notably, brevetoxin A (**25a**) contains one nine-member ring (Figure 7). They applied a five-step reaction sequence to BTXB (**25b**), forming derivatives that helped determine the absolute configuration. BTX is only found in dinoflagellates with a carbon skeleton composed of acetate and methyl acetate units. For the formation of the rings, there is a cascade mechanism from the epoxide groups in one end of the molecule to the other [1]. When the toxin enters the organism, it activates voltage-gated sodium channels, which causes a continuous sodium influx that depolarizes membranes and leads to repetitive firing in neurons [73]. Thus, brevetoxins possess neurotoxic and cytotoxic bioactivities. Given their biological activities, brevetoxins A (**25a**) and B (**25b**) affect fishing and tourism in areas where the dinoflagellates are found. These toxins cause neurotoxic shel-fish poisoning and kill fish. In addition, BTXs can lead to human food poisoning that causes gastrointestinal troubles and neurological disorders. Compared with other toxins in this family, brevetoxin A (**25a**) presents the highest toxicity potency [71].

Ciguatoxins C-CTX-1 (**26a**) and -2 (**26b**) (Figure 7) were isolated in a ratio of 2:1, respectively, from the horse-eye jack (*Caranx latus*), collected from St. Barthelemy in the Caribbean Sea [74]. A catfish sample was extracted and partially purified by mouse bioassay-directed fractionation. The toxic fractions were purified to homogeneity by RP-HPLC and analyzed by the MS of C-CTX-1 (**26a**) and C-CTX-2 (**26b**), obtained by ion spray MS (ISMS). C-CTX-1 (**26a**) and C-CTX-2 (**26b**) were subject to a mouse assay, and each toxin-induced signs that are typical of site-5 sodium channel activator toxins such as the Pacific ciguatoxins and brevetoxins [74]. One year later, the structure of **26a** and **26b** was reported, and they are composed of 14 transfused, ether-linked rings (A to N) containing a hemiketal in ring N (Figure 6) [75]. The 26a and 26b structures were determined based on the DQF-COSY, COSY, TOCSY, NOESY, ROESY, HSQC, and HMQC spectral data analysis. The relative stereochemistry of **26a,** like the all-*trans*-fused rings, *cis*-geometry of the double-bonds in the D, E, and F rings, and the orientation of the hydroxyl groups were determined from an analysis of *J* coupling constants and NOE data (Figure 7). The structural analysis of **26b**, the minor isomer, reveals that **26b** is the 56 epi-C-CTX-1. Marquais et al. reported studies where C-CTX-1 (**25a**) showed marked bradycardia in the mouse [76]. M. P. Sauviat et al. studied the mode of action **25a** on frog atrial heart muscle’s electrical and mechanical activity. They found that released acetylcholine (ACh) from atrial cholinergic nerve terminals activated the M1 and M2 muscarinic receptors subtype (mAChR) and started the Na^+^/Ca^2+^ exchange [77]. Caribbean ciguatoxins C-CTX-3 (**25c**) and -4 (**25d**) (Figure 7) were isolated from kingfish (*Scomberomorus cavalla)* and barracuda (*Sphyraena barracuda*), collected from St. Thomas, U.S. Virgin Islands, in 2014 and 2015 [78]. For ciguatoxin extraction, the fish were dissected, and, following toxicity assessment, highly toxic fish samples showing CTX-specific activity (>2.6 µg/kg C-CTX-3 equivalents) were selected for large-scale extraction. The toxicity evaluation of the models was performed by MTT assay with clonal mouse neuroblastoma (N2A) cells (MTT-N2A assay), and was used to measure mitochondrial activity in the presence of O/V. This assay identifies the presence of CTXs and studies sodium-channel-specific training to select the samples for further LC-HRMS/MS investigations. Extracts were homogenized, centrifugated, and evaporated to dryness, and residues were dissolved in methanol and screened by LC-MS. Extracts with the highest abundances of ciguatoxins **25a**, **25b**, **25c**, and **25d** (Figure 7) were chosen for further instrumental analyses and chemical reactions. Diagnostic product ions in LC-HRMS/MS were found for the ciguatoxins, and the analyses applied to the structure elucidation of new C-CTX congeners **25c** and **25d**. The combination of the MS data and the oxidation and reduction reactions provided enough information for the structure determination of new ciguatoxin analogs. They led to the definite structure determination of **25c** and **25d**. This was confirmed via reduction with NaBH_4_ and oxidation with NaIO_4_ of the analogs **25a** and **25b**, which then converted to **25c** and **25d**.

### 1.4. Unusual Polyketides

#### 1.4.1. Polyketides Containing Vinyl Chloride and Cyclopropane Moieties

**Figure 8:**
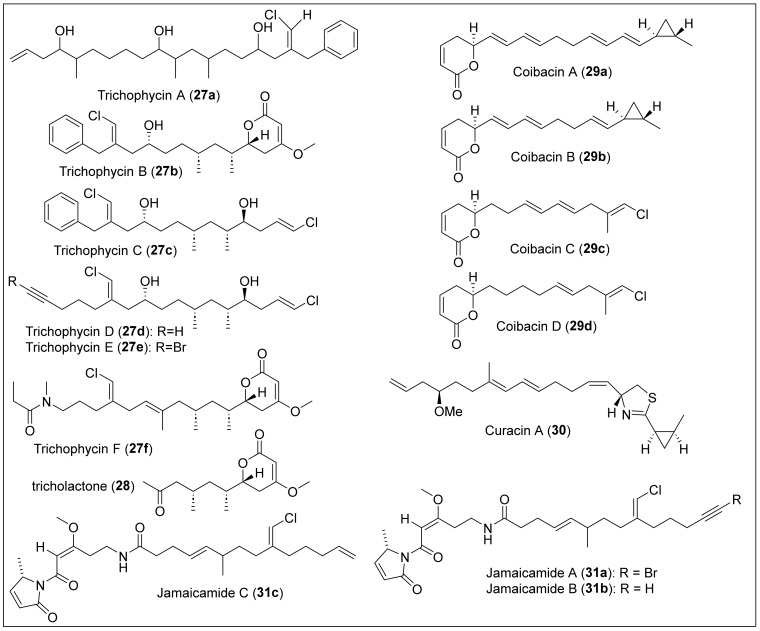
Structures of Caribbean hybrid polyketides **27**–**31**.

Trichophycin A (**27a**) (Figure 8) is a linear triol polyketide that was isolated in 2017 from *a Trichodesmium thiebautii* bloom in the Gulf of Mexico, specifically at Padre Island, Corpus Christi, Texas [79]. *Trichodesmium thiebautii* filaments were extracted with five separate portions and then fractionated over silica gel using vacuum liquid chromatography (VLC) using a stepped gradient. The resulting fractions were combined, based on similarities in ^1^H-NMR signals and similar potency in cytotoxicity assays, and then fractionated over a 2-g Strata C_18_ SPE column using a gradient. The most potent and cytotoxic fraction was subjected to RP-HPLC to isolate trichophycin A (**27a**). The structural elucidation was carried out by HRESIMS (C_29_H_47_ClO_3_), ^1^H and ^13^C-NMR and 2D NMR spectral data (HSQC, COSY, total correlation spectroscopy (TOCSY), HMBC and NOE). Trichophycin A (**27a**) has a planar structure, a vinyl chloride moiety, aromatic termini, and propionate units (Figure 8). The relative and absolute stereochemistry of **27a** remains undetermined. The cytotoxicity analysis (EC_50_) showed moderate capacity against the murine neuroblastoma Neuro-2A and HCT-116 cell lines, demonstrating (6.5 ± 1.4) µM and (11.7 ± 0.6) µM, respectively [79]. Five new highly functionalized polyketides, trichophycins B–F (**27b–f**) (Figure 8), and one non-chlorinated tricholactone metabolite (**27**) (Figure 8) were isolated from a collection of *Trichodesmium* bloom material from Padre Island, Corpus Christi, Texas [80]. The biomaterial was repeatedly extracted and isolated following the previously reported protocol to isolate trichophycin A (**27a**). The planar structures of trichophycins B–F (**27b–f**) were characterized by 1D and 2D NMR analysis, and HRESIMS determined the molecular formulas. The absolute configuration of **27b** and **27c** was determined with a modified Mosher esterification procedure, electronic circular dichroism (ECD) spectra, and *J*-based configuration analysis, supported by density functional theory (DFT) calculations. On the other hand, the absolute stereochemistry of **27d–f** and **28** was postulated based on the comparative values of ^13^C NMR chemical shifts, relative configurations, and optical rotation obtained for compounds **27b** and **27c** (Figure 8). The structure of compounds **27b**, **27f,** and **28** has a tricholactone, and the chlorovinylidene is present in compounds **27a–f**; the terminal vinyl chloride is observed in compounds **27c–e**, compound **27d** has a terminal alkyne, and compound **27e** has a unique alkynyl bromide functionality (Figure 8). Trichophycin E (**27e**) is a mixed polyketide–peptide with an *N*-methyl propanamide terminus. Cytotoxicity assays on mouse neuroblasts (Neuro-2A) revealed the toxicity of the isolated trichophycins and tricholactone. Trichophycin A (**27a**) has an EC_50_ (µM) of 6.5 ± 1.4. For **27b–f**, the EC_50_ (µM) is 14.8 ± 2.4, 23.8 ± 4.2, 39.8 ± 3.8, low toxicity even at 100 μM, and 14.3 ± 2.3 μM, respectively.

Coibacins A–D (**29a**–**d**) (Figure 8) were isolated from Panamanian filamentous marine cyanobacteria cf. *Oscillatoria*, collected in Panama [81]. These metabolites are classified as linear unsaturated polyketides that contain an α,β-unsaturated δ-lactone (Figure 8). There are two types of termini for the unsaturated chain in coibacins, which are the *trans*-methyl-substituted cyclopropyl ring in coibacins A (**29a**) and B (**29b**), and a methyl vinyl chloride with trans configuration in coibacins C (**29c**) and D (**29d**), which are very similar to the structure motif in curacin A (**30**) [82] and jamaicamide A (**31a**) (Figure 8) [83]. Coibacins (**29a**–**d**) (Figure 7) have different unsaturated chains with varying amounts of trans double bonds. In the isolation procedure, the extract of cf. *Oscillatoria* sp. was pre-fractioned using CH_2_Cl_2_/MeOH solvents. Then, the fractions were purified using reversed-phase solid-phase extraction (RP-SPE) followed by RP-HPLC with C_18_. After assays, coibacins possessed antileishmanial and potent anti-inflammatory activity. Coibacins (**29a**–**d**) presented action against axenic amastigotes of *Leishmania donovani* (IC_50_ = 2.4 μM), where coibacin A (**29a**) had the highest activity. However, these metabolites did not present activity against *L. mexicana* macrophages, malaria, and Chang’s disease. Cytotoxicity assays against NCI-H460 human lung cancer cells demonstrated that coibacin A (**29a**) had the lowest potency, with an IC_50_ value of 11.4 μM, while coibacin D (**29d**) had the highest (IC_50_ = 31.5 μM). Coibacin B (**29b**) was the most active (IC_50_ = 5 μM) for anti-inflammatory activity in a cell-based nitric oxide (NO) inhibition assay. Using the enzyme-linked immunosorbent assay, coibacins (**29a**–**d**) reduced TNF-α and IL-6 secretion, indicating changes in protein expression of inflammatory cytokines, with the highest potency from coibacin A (**29a**) at 10 μg/mL.

Curacin A (**30**) (Figure 8) was isolated by Gerwick et al. from the marine cyanobacterium *Lyngbya majuscula*, discovered in Curacao [82]. It is a polyketide synthase (PKS)–non-ribosomal peptide synthetase derived natural product from two polyketides joined together through a decarboxylated L-cysteine residue [37]. Its isolation was achieved through bioassay-guided fractionation based on brine shrimp toxicity [38]. The sample of *L. majuscula* was extracted by two consecutive rounds of vacuum chromatography to obtain a crude sample of curacin A (**30**) that was HPLC purified. HRFABMS confirmed the molecular formula of C_23_H_35_NOS. Initially, the planar structure of curacin A (**30**) was determined by NMR spectroscopy studies (^1^H and ^13^C NMR, COSY, HMBC, and NOE), which confirmed the substitution pattern and geometry of the olefins and cyclopropane ring, and the presence of the thiazole ring (Figure 8) [82]. Later, it was reported that curacin A (**30**) has a 2*R*, 13*R*, 19*R*, and 21*S* absolute configuration, established by comparing the chemical degradation products with the same compounds prepared by synthesis [84]. Curacin A (**30**) inhibits tubulin polymerization and shows inhibitory activity against L1210 leukemia cells (IC_50_ = 9 × 10^−9^ M) and CA46 Burkitt lymphoma cells IC_50_ = 2 × 10^−7^ M). Further bioactivity assays revealed that compound **30** is a potent inhibitor of MCF-7 breast cancer cell growth and mitosis [85].

Jamaicamides A–C (**31a**–**c**) (Figure 8) are highly functionalized hybrid polyketide–peptide neurotoxins isolated from *Lyngbya majuscule*, obtained at Jamaica’s Hector’s Bay [83]. Organisms such as marine cyanobacteria (blue–green algae) have a metabolism responsible for two mechanisms. The first mechanism is the biosynthesis of compounds from nonribosomal peptide synthetases (NRPSs), such as amino acids. The other mechanism is polyketide synthases, to generate compounds derived from the acetate pathway. Jamaicamides A–C (**31a**–**c**) result from predicting the alternate working of PKS and NRPS. These compounds demonstrate sodium channel blockade and toxicity in fish. Marine cyanobacteria bioactive metabolites can attack the polymerization of actin and tubulin and affect the voltage of sodium channels by blocking or activating them. The extraction of jamaicamides A–C (**31a**–**c**) from the sample of *L. majuscule* was performed with standard methods for drawing lipids. First, they were extracted with dichloromethane:methanol (2:1) to obtain a crude extract. Then, a TLC performed with hexane/EtOAc determined the presence of the lipids. Later, vacuum liquid chromatography (VLC) was performed, and, to obtain the new lipopeptides, TLC, ^1^H-NMR, and HPLC were performed. Spectral data from COSY, ^1^H and ^13^C NMR, HSQC, HSQC-COSY, and HMBC were analyzed to determine the structures of **31a**–**c**. In addition, the sample’s UV-Vis and IR spectroscopic studies were performed. The polyketide moiety in jamaicamides A–C (**31a**–**c**) contains a trisubstituted (*E*)-chloro olefin, an undetermined methyl stereocenter (C_9_), an (*E*)-olefin (C_10_–C_11_), a pyrrolidine ring, and a β-methoxy enone. Jamaicamide A (**31a**) has an unusual alkynyl bromide, and jamaicamide B (**31b**) and C (**30c**) have a terminal alkyne and alkene, respectively (Figure 8). Jamaicamide B (**31b**) was isolated as a yellow oil and is slightly more polar than jamaicamide A (**31a**), which makes it elute earlier than **31a** under RP-HPLC conditions. Jamaicamide C (**31c**) was isolated in small amounts (0.5% crude extract) as a yellow oil and is slightly more hydrophobic than **31a** and **31b**. Jamaicamide A (**31a**) has an unusual structure, an alkynyl bromide (Figure 8). All three compounds have a vinyl chloride at the center of an extended polyketide chain, a rare occurrence (Figure 8). In mice, jamaicamides (**31a–c**) exhibited cytotoxic properties against human neuroblastoma-lung (H-460) and Neuro-2a. The adequate amount for compounds **31a–c** was 15 μM, and blocked sodium channels at 5 μM; none activated sodium channels. A cytotoxicity study for 90 min with goldfish found that jamaicamide B (**31b**) was 100% lethal at 5 ppm, jamaicamide C (**31c**) was 100% lethal at 10 ppm, and jamaicamide A (**31a**) was sublethal at 10 ppm. The total synthesis of jamaicamides A–C (**31a**–**c**) has not been accomplished, but the stereoselective nonracemic synthesis of the polyketide chain of jamaicamide C (**31c**) was reported in 2009 by Graf and coworkers [86]. Later, S. Watanabe et al. reported the synthesis of the polyketide (*E*)-olefin of the jamaicamides [87]. 

**Figure 9:**
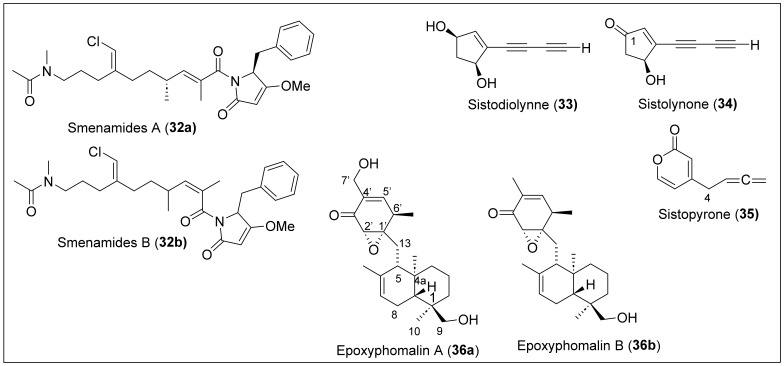
Structures of Caribbean hybrid polyketides **32**–**36**.

Smenamides A (**32a**) and B (**32b**) (Figure 9) are hybrid peptide/polyketide compounds that have been isolated from the Caribbean sponge *Smenospongia aurea*, collected at the coast of Little Inagua, Bahamas Islands [88]. The compound was isolated by extracting with methanol and separated by flash chromatography on silica gel, followed by reversed- and normal-phase chromatography. The complete assignment of the planar structures of **32a** and **32b** was elucidated using homo- and heteronuclear 2D NMR experiments (COSY, TOSCY, ROESY, HSQC, and HMBC) and by high-resolution ESI-MS/MS. Compounds **32a** and **32b** are *E/Z* isomers containing vinyl chloride, dolapyrrolidinone, and *N-dimethylacetamide* units, as shown in Figure 9. Smenamides (**32a**) and (**32b**) were tested in vitro for lung cancer cells from the NSCLC cell line and showed positive cytotoxic effects at concentrations of 50 nM. Smenamides A (**32a**) and B (**32b**) showed an IC_50_ of 48 nM and 49 nM, respectively. Similar results were achieved with solid tumors such as breast, ovary, and melanoma cancer cell lines.

#### 1.4.2. Norpentaketides

During the inspection of the air quality of a museum in Puerto Rico, a wood-decay fungus was discovered with the name of *Sistotrema raduloides* Donk, from which multiple unusual polyketides were extracted [89]. The compounds extracted were named sistodiolynne (**33**), sistolynone (**33**), and sistopyrone (**34**) (Figure 9). These compounds are known as norpentaketides, meaning that the methyl carbon of one of the acetate units has been lost. For extraction, *S. raduloides* was grown on Sabouraud dextrose broth, and filtration was used to separate the mycelium. Then the filtrate was extracted with CH_2_Cl_2_ and then with ethyl acetate. Sistodiolynne (**33**) (C_9_H_8_O_2_), sistolynone (**34**) (C_9_H_6_O_2_), and sistopyrone (**35**) (C_10_H_9_O_2_) were purified and obtained via preparative TLC. NMR, IR, and UV measurements provide the spectral data to determine the structures of **33**, **34**, and **35**. Sistodiolynne (**33**) has a base structure of a 4-cyclopentene with two alcohol substituents in carbon 1 and 3 and a conjugated enediyne on carbon 4 (Figure 6). Sistolynone (**34**) has a similar structure; the only difference is the presence of a ketone at C_1_ (Figure 9). The third compound, **35**, contains a six-membered ring known as a pyrone, with an allene group substituent on carbon 4 (Figure 7). During the extraction process, it was found that the compounds are unstable, and, because of this, no biological activity can be investigated [80].

#### 1.4.3. Prenylated Polyketides

Epoxyphomalins A (**36a**) and B (**36b**) (Figure 9), prenylated polyketides, were isolated from marine-derived filamentous fungi, the facultative marine fungus *Phoma* sp. [81], obtained from the marine sponge *Ectyplasia perox*, collected from the Caribbean Sea outside Dominica. HREIMS measurements determined that the molecular formula for epoxyphomalin A (**36a**) is C_20_H_32_O_5_, and C_20_H_32_O_4_ for epoxyphomalin B (**36b**). Structural characterization of **36a**–**b** was carried out by IR and 1D and 2 NMR analysis. Compounds **36a**–**b** are composed of a *trans*-decalin ring system connected to a cyclohexanone ring with an epoxide attached to it (epoxydon moiety) (Figure 9). The difference between **36a** and **36b** is in the C-7′ part of the cyclohexanone ring. The difference can be explained by the presence of methyl in epoxyphomalin B (**36b**) instead of the hydroxylated methylene group found in epoxyphomalin A (**36a**). The relative configuration of the decalin portion for **36a**–**b** is 1*R*, 4*R*, 5*S*, and 8*R*, and the absolute configuration of the epoxide is 1′*S* and 2′*S* (Figure 7). For the isolation and purification, the fungus was cultivated, and the successive fractionation of the crude EtOAc extract of the fungal mycelium and medium by chromatography on silica and Sephadex LH-20 material, followed by RP HPLC, yielded compounds **36a** and **36b**. The cytotoxic effects of epoxyphomalins (**36a**) and (**36b**) were investigated using a monolayer cell survival and proliferation assay in a panel of 36 human tumor cell lines comprising 14 different solid tumor types, and both compounds were found to be active. Epoxyphomalin (**36a**) showed superior cytotoxicity at nanomolar concentrations toward 12 of the 36 human tumor cell lines, such as breast cancer MAXF 401NL and adeno lung cancer LXFA 629 L. The observed cytotoxic selectivity pattern of epoxyphomalin A (**36a**) did not correlate with those of reference anticancer agents with known mechanisms of action. The cytotoxicity studies of epoxyphomalin B (**36b**) showed IC_50_ values in cell lines such as pleura mesothelioma PXF 1752 L (0.251 μg/mL) and bladder cancer BXF T24 (0.402 μg/mL).

## 2. Conclusions

The polyketides isolated and studied in this report came from Caribbean microorganisms, including marine sponges, corals, cyanobacteria, dinoflagellates, mollusks, and certain types of algae. These isolated Caribbean secondary metabolites have promising bioactive properties, such as antimicrobial, anticancer, anti-inflammatory, and antiviral activities that are demonstrated to be potential candidates for developing new drugs.

From the 90 Caribbean polyketides summarized, we found three main subgroups of polypropionates, which include macrolides, and linear and polycyclic ethers (Figure 4, Figure 5, Figure 6, Figure 7, Figure 8 and Figure 9) (Table 1). Furthermore, a group of mixed or hybrid polyketides that have uncommon functional groups were also included and discussed (Figure 7). The absolute stereochemistry of most of the reviewed Caribbean polyketides has been well determined and reported. Nevertheless, the absolute configurations of the groups caribenolide I (**7**) and prorocentrolide B (**9**) macrolactones (Figure 3), poecillastrins A–C (**10a**–**c**) macrolactams (Figure 4), caylobolides A–B (**11a**–**b**) macrolide polyols (Figure 4), trisphaerolide A (**16**), hyapyrone B (**17**) and amphidinol 17 (**19**) linear polyketides (Figure 5), and the hybrid polyketides trichophycin A (**27a**), jamaicamides A–C (**31a**–**c**) and smenamide B (**32b**) (Figure 7) have not been determined up till now. On the other hand, looekeyolides A–D (**4a**–**d**) (Figure 3), smenopyrone (**18**) (Figure 5), and norpentaketides (**33**–**35**) (Figure 7) are the only polyketides that either do not show bioactivity or are not reported. Places such as Puerto Rico, the Virgin Islands, the Bahamas, Belize, Florida, Dominica, and St. Thomas, among others, are well represented by the biodiversity of organisms that produce polyketides around these tropical Islands (Table 1). These secondary metabolites hold great potential for drug discovery, as they often possess unique chemical structures and bioactivities. There are still pending efforts to review some of the compounds’ structural elicitations and biological activity. By studying and harnessing the power of these compounds, scientists aim to develop new therapeutic agents and gain further insight into the ecological role of polyketides in marine ecosystems. With this review, the future perspective is to motivate researchers to continue exploring the Caribbean region’s marine and terrestrial environments to revisit, discover and investigate new bioactive polyketide and polypropionate natural products.

## Figures and Tables

**Table 1:** Summary of polypropionate and polyketide natural products by location in the Caribbean region.

Caribbean Region	Natural Product	Reference
Summerland Key, Florida	Tedanolide (**2**)	[16]
Key West, Florida	Caylobolide B (**11b**)	[41]
Naples, Florida, Gulf of Mexico	Lasonolide C–G (**3c**–**g**)	[22]
Florida, Gulf of Mexico	Bryostatin 16–18 (**8a**–**c**)	[31,32]
Florida, Gulf of Mexico	Brevetoxin A (**25a**)	[71]
Florida, Gulf of Mexico	Brevetoxin B (**25b**)	[72]
Padre Island, Corpus Christi, Texas, Gulf of Mexico	Trichophycin A (**27a**)	[79]
Padre Island, Corpus Christi, Texas, Gulf of Mexico	Trichophycin B–F (**27b**–**f**) and Tricholactone (**28**)	[80]
Florida, Belize, and Honduras	Looekeyolides A–B (**4a**–**b**)	[23]
Florida and Belize	Looekeyolide C–D (**4c**–**d**)	[24]
Belize	Lobophorin A–B (**12a**–**b**)	[38,39]
Dauphin Island, Alabama	Prorocentrolide B (**9**)	[37]
Bahamas	Lobophorolide (**5**)	[25]
Grand Bahama Island, Bahamas	Poecillastrin A–C (**10a**–**c**)	[34,35]
Bahamas	Caylobolide A (**11a**)	[40]
Bahamas	Bahamaolides A–B (**13a**–**b**)	[50]
Bahamas	Smenopyrone (**18**)	[58]
Little San Salvador Island, Bahamas	Amphidinol 17 (**19**)	[59]
Little Inagua, Bahamas	Smenamides A–B (**32a**–**b**)	[88]
St. Thomas, U.S. Virgin Islands	Amphidinolide B_1–3_ (**6a**–**c**)	[25,26,27]
St. Thomas, U.S. Virgin Islands	Caribenolide I (**7**)	[31]
St. Thomas, U.S. Virgin Islands	Ciguatoxins-3 (**26c**) and -4 (**26d**)	[78]
British Virgin Islands	Lasonolide A (**3a**)	[18,19]
British Virgin Islands and Florida Keys	Okadaic Acid (**22**)	[59,60]
Cuba	Dolabriferol (**15a**)	[54]
Puerto Rico	Dolabriferol B–C (**15b**)	[55]
Puerto Rico	Aplysqualenol A–B (**24a**–**b**)	[70]
Puerto Rico	Sistodiolynne (**33**), Sistolynone (**34**) and Sistopyrone (**35**)	[89]
Jamaica	(+)-Neopeltolide (**1**)	[12]
Jamaica	Jamaicamides A–C (**31a**–**c**)	[83]
Dominica	Trisphaerolide A (**16**)	[56]
Dominica	Spirastrellolide A–G (**20a**–**g**)	[55,56,57,58]
Dominica	Epoxyphomalin A–B (**36a**–**b**)	[90]
Windward Islands	Hyapyrone B (**17**)	[57]
Martinique	Lucensimycin A–G (**23a**–**g**)	[61,62,63]
St. Barthelemy	Ciguatoxins-1 (**26a**) and -2 (**26b**)	[68,69]
Curacao	Curacin A (**30**)	[82]
Santa Marta, Colombia	(+)-Discodermolide (**14**)	[51]
Panama	Coibacin A–D (**29a**–**d**)	[81]

## Data Availability

The data are contained within the article.
